# Unrepaired ramp lesions are associated with a higher risk of secondary medial meniscus bucket handle tear compared to lateral meniscus bucket handle tear after anterior cruciate ligament reconstruction

**DOI:** 10.1002/jeo2.70176

**Published:** 2025-02-24

**Authors:** Alexander J. Hoffer, Ahmed Mabrouk, Christophe Jacquet, Matthieu Ollivier

**Affiliations:** ^1^ Department of Orthopaedics University of British Columbia Vancouver British Columbia Canada; ^2^ Trauma & Orthopaedics Department Leeds Teaching Hospitals Leeds UK; ^3^ Institut du movement et de l'appareil locomoteur Marseille France

**Keywords:** ACL reconstruction, anterior cruciate ligament, bucket handle tear, meniscus, ramp

## Abstract

**Purpose:**

To compare the risk of a secondary bucket handle tear (BHT) of the medial and lateral menisci after an anterior cruciate ligament reconstruction (ACLR) with an unrecognized ramp lesion. The hypothesis was that an unrecognized ramp lesion would be associated with a secondary medial meniscus BHT more often than a lateral meniscus BHT.

**Methods:**

A retrospective review of adults aged 18 or older who experienced a meniscal BHT after ACLR was conducted. An analysis of the clinical and radiological data from initial injury to revision surgery was completed. Two experts retrospectively documented the prevalence of ramp lesions present on preoperative magnetic resonance imaging (MRI) at the time of the index ACLR. The predictive value of a ramp lesion for BHT laterality was evaluated using logistic regression.

**Results:**

Seventy‐six patients, 46 in the medial BHT group and 30 in the lateral BHT group, were included. A ramp lesion was present on the preoperative MRI in 33 patients in the medial BHT group compared to 13 in the lateral BHT group (*p* = 0.02, odds ratio: 3.2, 95% confidence interval: 1.2–8.0). In the logistic regression analysis, the only independent factor that predicted the occurrence of a medial BHT compared to a lateral BHT was the presence of a ramp tear on preoperative MRI before the index ACL surgery (logworth = 1.59; *p* = 0.03).

**Conclusion:**

After a primary ACLR, an untreated ramp lesion was associated with a post‐operative medial BHT more often than a lateral BHT. Unrepaired ramp lesions may be a risk factor for subsequent medial meniscus BHT after primary ACLR.

**Level of Evidence:**

Level IV.

**Abbreviations**
ACL: anterior cruciate ligament
ACLR: anterior cruciate ligament reconstruction
ASA: American Society of Anesthesiologists
BHT: bucket handle tear
BMI: body mass index
IKDC: International Knee Documentation Committee
MLKI: multi‐ligament knee injury
MRI: magnetic resonance imaging
PLT: posterior horn or root tear
PRO: patient‐reported outcome
ROM: range of motion
SKV: Simple Knee Value
UCLA: University of California and Los Angeles

## INTRODUCTION

Concomitant meniscal injury during anterior cruciate ligament (ACL) rupture occurs between 47% and 61% of the time [1, 16]. While the lateral meniscus is more commonly damaged during an acute ACL injury, once an individual has experienced more than one episode of recurrent instability, longitudinal posterior horn medial meniscal tears become the most common meniscal injury pattern [15, 22, 26]. Ramp lesions are a previously underrecognized meniscal injury described as meniscus‐synovial, meniscocapsular or meniscotibial detachment with a resultant peripheral vertical longitudinal tear at the peripheral posterior horn of the medial meniscus [8, 20, 44]. The prevalence of ACL injury with concomitant ramp lesions ranges from 9.3% to 39.5% [6, 9, 28].

Despite the improved awareness of ramp lesions, their diagnosis may still be underestimated for several reasons. First, visualization of a ramp tear may be difficult on preoperative magnetic resonance imaging (MRI), with a reported sensitivity of 48% to 86% [7]. Second, standard arthroscopic assessment from the anterolateral viewing portal has low sensitivity due to the inadequate visualization of the peripheral posterior horn of the medial meniscus from the medial compartment of the knee [23]. Third, a trans‐notch view of the posteromedial meniscocapsular junction with a 30° arthroscope may not visualize the lateral aspect of the junction and result in missed ramp lesions [23]. Finally, ramp lesions may be hidden underneath a thin layer of membrane‐like tissue that is only accessible through a posteromedial portal [42].

A meniscus bucket handle tear (BHT) is a longitudinal or oblique vertical tear with an unstable inner component and intact peripheral attachments anteriorly and posteriorly [46]. Displacement of the inner component between the weight‐bearing surfaces or into the notch may result in mechanical symptoms and pain [37, 39]. Repair of a meniscus BHT is important to maintain medial and lateral compartment contact pressures and avoid secondary osteoarthritis [21, 30]. Thaunat et al. previously reported an association between unrepaired ramp lesions and secondary meniscus BHT. They identified six medial meniscus BHTs at 8–10 years after ACL reconstruction (ACLR) in 28 patients who had unrepaired stable ramp lesions at the time of initial reconstruction [45]. However, the relationship between ramp lesions and meniscal BHT laterality has not been previously explored.

Ramp lesions are associated with increased anterior tibial translation, a higher grade of dynamic rotational laxity and excessive knee rotational mobility in the context of an ACL injury [32, 33]. However, only one previous study reported an association between an untreated ramp lesion during an ACLR and subsequent medial meniscal BHT. No study has considered the relationship between unrepaired ramp lesions and secondary meniscal BHT laterality. The purpose of this study was to further delineate the relationship between an unrecognized ramp lesion during ACLR and subsequent BHT meniscus injuries in the knee. The hypothesis was that an unrecognized ramp lesion would be associated with a secondary medial meniscus BHT more often than a lateral meniscus BHT.

## METHODS

### Patient selection, treatment and outcome measures

After institutional review board approval was obtained (PADS21‐177_dgr), a retrospective review was performed between January 2016 and December 2021 for all patients who underwent meniscus surgery at a single institution. The inclusion criteria were adults aged 18 years or older who sustained a meniscal BHT following an ACLR and had an available preoperative MRI before ACLR. Exclusion criteria were incomplete clinical or radiological data, no ACLR, a meniscal BHT before the ACL injury, multi‐ligament knee injury, concomitant osteotomy, revision ACLR or present systemic inflammatory disease affecting healing.

In this study, two orthopaedic surgeons with sports knee subspecialty training from a single institution contributed to the primary ACLR and secondary meniscus surgery. The surgeries were performed using standardized arthroscopic‐assisted ACLR techniques. The index ACLR graft type, meniscal injury presence, laterality, location, pattern, size and subsequent treatment were recorded. The completion of a trans‐notch view and the presence or absence of a ramp lesion were recorded when performed intra‐operatively. Meniscal injuries were treated based on their injury characteristics according to the regional standard of care. In general, meniscal repair was completed unless the tear was irreparable or lacked any healing potential. The prevalence and type of a concomitant lateral extra‐articular procedure at the time of index ACLR was recorded.

Clinical and radiological data from the initial injury to the revision surgery were retrieved. Patient demographics, including age, height, weight, body mass index (BMI), laterality of affected knee, American Society of Anesthesiologists (ASA) score, smoking status, index ACL, sports participation and competition level before and after ACLR, and affected lower extremity mechanical axis, were recorded. A fellowship‐trained musculoskeletal radiologist and knee‐subspecialized orthopaedic surgeon independently assessed the blinded preoperative MRI of included patients and documented the presence of a ramp lesion based on fluid‐filling hyperintensity between the medial meniscus posterior horn and capsule and secondary posteromedial tibial bone bruise [13, 27, 43]. Any disagreements were discussed between the two investigators, and if agreement could not be obtained, a second orthopaedic surgeon independently assessed the blinded MRI to achieve a majority decision. Concomitant meniscal and cartilage injuries at initial and subsequent surgery, including a description of the ramp lesion present during revision surgery, were recorded [43]. Post‐operative meniscal BHTs were separated into a medial BHT group and a lateral BHT group, and the interval between initial ACLR and subsequent management (repair or excision) was recorded.

The University of California and Los Angeles (UCLA) patient‐reported outcome (PRO) score was recorded at the time of initial consultation for all patients preoperatively. Post‐operatively, the UCLA, Lysholm, International Knee Documentation Committee score (IKDC) and Simple Knee Value (SKV) scores were recorded at post‐operative in‐person or telehealth follow‐up visits [3, 19, 24, 31].

### Post‐operative care

A brace was not routinely used post‐operatively. Weight‐bearing and range of motion (ROM) as tolerated with crutches were permitted for 2 weeks. At 2 weeks post‐operatively, the patient was weaned off crutches and began physiotherapy with a focus on ROM. The only variable that changed the post‐operative protocol was the presence of a meniscal root repair. If a root repair was completed, the patient was placed in a brace locked in extension for 2 weeks post‐operatively. The brace was unlocked after 2 weeks and worn for 6 weeks in total. Patients were permitted toe‐touch weight bearing for the first 6 weeks post‐operatively.

Athletes went through several tests before being cleared for return to sport, with identical protocols between groups. In addition to full ROM and no effusion, quadriceps and hamstring strength were required to be at least 90% of the non‐operative side. In addition, the single hop and triple crossover hop test for distance had to be within 10% of the non‐injured leg. Finally, the athlete completed lower extremity functional testing with their physical therapist. They then advanced to sports‐specific training increasing activities to full contact under athletic trainer supervision. Final clearance came from the senior surgeon when the patient returned to practice and displayed no hesitancy or compensation strategies during cutting drills, especially deceleration at full effort.

### Statistical analysis

Statistical analysis was performed using computation software (JMP 17). Patient demographics and surgical details were recorded. A chi‐square test or Fisher's exact test was performed to compare categorical variables between the medial and lateral BHT groups depending on their distributions. Independent *t* tests or Mann–Whitney *U* tests were performed to compare continuous variables between the medial and lateral BHT groups depending on their distributions. The inter‐observer correlation coefficient was calculated to ensure the accuracy of diagnosing a ramp lesion on preoperative MRI. The predictive value of a ramp lesion on preoperative MRI for a post‐operative meniscal BHT was evaluated using logistic regression, adjusted for potential confounders.

## RESULTS

### Baseline characteristics

**Figure 1:**
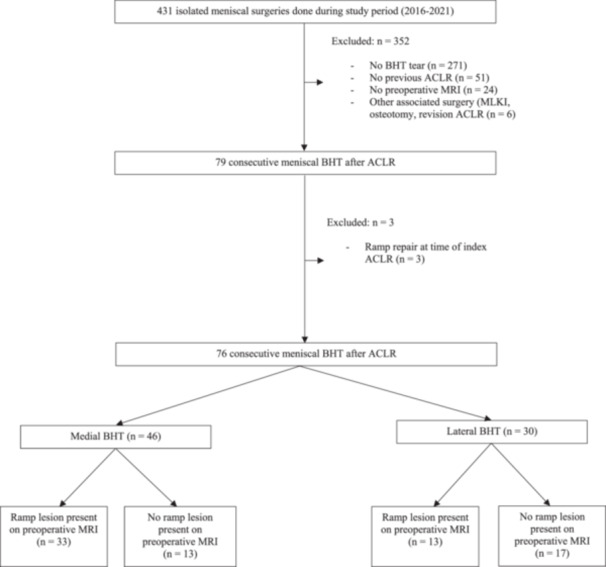
Flowchart displaying patient selection for this study. ACLR, anterior cruciate ligament reconstruction; BHT, bucket handle tear; MRI, magnetic resonance imaging; MLKI, multi‐ligament knee injury.

About 431 patients underwent isolated meniscal surgery during the study collection dates. Seventy‐six patients, 15 females, were included in the final analysis (Figure 1). Overall, 1375 ACLRs were completed during the study collection dates, and the BHT complication rate was 5.5%. Baseline characteristics are summarized in Table 1. The average time between the initial ACL injury and revision surgery was 24 ± 6 months in the medial BHT group and 26 ± 7 months in the lateral BHT group (*p* = 0.50).

**Table 1:** Patient characteristics.

Variable	Total	Medial BHT	Lateral BHT	*p* values
Gender				*p* = 0.2
Male	61	35 (75%)	26 (87%)	
Female	15	11 (25%)	4 (13%)	
Age	31.1 ± 10	31.8 ± 9	30.4 ± 11	*p* = 0.9
Weight (kg)	73 ± 10.5	74.5 ± 12.8	72 ± 5.7	*p* = 0.8
Height (m)	1.74 ± 0.08	1.72 ± 0.08	1.78 ± 0.05	*p* = **0.0005**
Side				*p* = 0.1
Right	29	15	14	
Left	57	31	16	
ASA				*p* = 0.7
I	71	43	28	
II	5	3	2	
Smoking status				*p* = 0.4
Non‐smoker	53 (69%)	29 (63%)	24 (80%)	
Smoker	19 (25%)	14 (30%)	7 (20%)	
Missing data	4 (6%)	4 (7%)	0 (0%)	
HKA (°)	178.6 ± 3	179 ± 1	177.9 ± 1.4	*p* = 0.3

*Note*: bold, significant *p* < 0.05.

Abbreviations: ASA, American Society of Anesthesiologists; BHT, bucket handle tear; BMI, body mass index; HKA, hip–knee–ankle.

### MRI findings and surgical status

A ramp lesion was diagnosed in 33 out of 46 patients in the medial BHT compared to 13 out of 30 in the lateral BHT group (*p* = 0.02, odds ratio: 3.3, 95% confidence interval: 1.3–9) retrospectively on MRI. A preoperative lateral meniscus posterior horn or root tear (PLT) was diagnosed in 18 patients in the medial BHT group and 11 in the lateral BHT group (*p* = 0.40). Initial injury, index ACLR and revision surgery details are presented in Table 2. PLT repair was performed in three patients in the medial BHT group and two patients in the lateral BHT group (*p* = 0.60).

**Table 2:** Original injury, index anterior cruciate ligament reconstruction (ACLR) and revision surgery details.

Specifics of injury and index surgery	Total (%)	Medial BHT (%)	Lateral BHT (%)	*p* value
Ramp lesion retrospectively identified on MRI	46 (60.5)	33 (71.7)	13 (43.3)	0.02
Description of ramp lesion				0.003
Meniscocapsular lesion in synovial sheath	7 (15.2)	2 (6.1)	5 (38.5)	
Partial superior lesion	11 (23.9)	10 (30.3)	1 (7.7)	
Partial inferior lesion	6 (13.0)	6 (18.2)	0 (0)	
Complete red/red tear	16 (34.8)	9 (27.3)	7 (53.8)	
Complete double tear	6 (13.0)	6 (18.2)	0 (0)	
Other meniscus injury				0.5
None	61 (80.2)	38 (82.6)	23 (76.7)	
Medial meniscus	7 (9.2)	4 (8.7)	3 (10)	
Lateral meniscus	8 (10.5)	4 (8.7)	4 (13.3)	
ACLR graft				0.7
Hamstrings	35 (46.1)	21 (45.6)	14 (46.7)	
Hamstrings + LET	13 (17.1)	9 (19.6)	4 (13.3)	
BTB	19 (25)	11 (23.9)	8 (26.7)	
BTB + LET	9 (11.8)	5 (10.9)	4 (13.3)	

Specifics of the revision surgery	Total (%)	Medial BHT (%)	Lateral BHT (%)	*p* value
ACLR status				0.7
Intact graft	37 (48.7)	23 (50)	14 (46.7)	
Loose graft but competent	16 (21.1)	11 (23.9)	5 (16.7)	
Loose graft and abnormal	23 (30.2)	12 (16.1)	11 (36.7)	
ACLR revision status				0.7
Not revised	40 (52.6)	23 (50)	17 (56.7)	
Revised	36 (47.4)	23 (50)	13 (43.3)	
Revision ACLR graft				0.9
Not revised	40 (52.6)	23 (50)	17 (56.7)	
Bone‐patellar tendon‐bone	1 (2.8)	1 (2.2)	0 (0)	
Quadriceps tendon	22 (28.9)	12 (26.1)	10 (33.3)	
Contralateral hamstring	5 (6.6)	3 (6.5)	2 (6.7)	
Allograft	3 (3.9)	2 (4.3)	1 (3.3)	
Missing data	5 (6.6)	5 (10.9)	0 (0)	
Average time to BHT (months)	25 ± 6	24 ± 6	26 ± 5	0.9
BHT management				0.8
Meniscectomy	33 (43.4)	20 (43.5)	13 (43.3)	
Repair	43 (56.4)	26 (56.5)	17 (56.7)	
All‐inside	25 (58.1)	13 (50)	12 (70.6)	
All‐inside and outside‐in	12 (27.9)	7 (26.9)	5 (29.4)	
All‐inside and inside‐out	4 (9.3)	4 (15.4)	0 (0)	
All‐inside, inside‐out and outside‐in	2 (4.7)	2 (7.7)	0 (0)	
Cartilage lesions				0.8
No cartilage lesions	44 (57.9)	26 (56.6)	18 (60)	
Associated cartilage lesions	32 (42.1)	20 (43.5)	12 (40)	
Medial collateral ligament (MCL) status				0.04
No MCL injury	70 (91.1)	40 (85.4)	30 (100)	
MCL injury	6 (8.9)	6 (14.6)	0 (0)	
Associated procedures				0.1
No other surgeries	58 (76.3)	35 (76.1)	23 (76.7)	
Cartilage surgery	8 (10.5)	3 (6.5)	5 (16.7)	
Micro‐drilling	6 (75)	3 (50)	3 (60)	
Mosaicplasty	2 (25)	0 (0)	2 (40)	
Meniscal debridement	10 (13.2)	8 (17.4)	2 (6.7)	

Abbreviations: BHT, bucket handle tear; BTB, bone‐patellar tendon‐bone; LET, lateral extra‐articular tenodesis; MRI, magnetic resonance imaging.

### Logistic regression

In the logistic regression analysis, no potential confounding factors, including sex, BMI, HKA, ACLR specifics, return to sport, competition level or PRO scores, were predictive of a medial BHT over a lateral BHT (all *p* > 0.05, Table 3). The only variable that predicted a medial BHT compared to a lateral BHT was the presence of a ramp lesion on preoperative MRI before the index ACLR (logworth = 1.7; *p* = 0.03).

**Table 3:** Coefficients of the logistic regression model.

Variable	Logworth	*p* value
Sex	0.04	0.90
BMI	0.18	0.66
Age	0.30	0.51
HKA	0.001	1
ACLR graft	0.3	0.5
BHT laterality	**1.7**	**0.03**
Return to sport	0.01	0.98
Competition level	0.10	0.79
PRO score:UCLA	0.008	0.98

*Note*: bold, significant *p* < 0.05.

Abbreviations: ACLR, anterior cruciate ligament reconstruction; BHT, bucket handle tear; BMI, body mass index; HKA, hip–knee–angle; PRO, patient‐reported outcome; UCLA, University of California and Los Angeles.

### Outcomes

There was no difference in the return to sport, competition level or preoperative UCLA scores between the medial and lateral BHT groups (Table 4). Similarly, there was no difference in the post‐operative clinical outcome scores including the Lysholm, IKDC, UCLA and SKV between the medial and lateral BHT groups at a minimum of 2 years follow‐up (Table 4).

**Table 4:** Preoperative and post‐operative outcome scores.

Sports specifics	Total (%)	Medial BHT	Lateral BHT	*p* value
Preoperative assessment				
Sports activity				0.9
No sports	6 (8.9)	4 (8.3)	2 (9.7)	
Active sports	70 (91.1)	42 (91.7)	28 (90.3)	
Activity rate (h/week)	6.5 ± 7.1	5.8 ± 6.7	7.7 ± 7.8	0.1
Sports at competition level				0.5
Competition level	35 (45.6)	20 (41.7)	15 (51.6)	
Non‐competition level	41 (54.4)	26 (58.3)	15 (48.4)	
UCLA knee score				0.01
6 (% of participants)	7 (8.1)	2 (4.2)	5 (19.4)	
7 (% of participants)	18 (22.8)	13 (27.1)	5 (16.1)	
8 (% of participants)	16 (22.8)	12 (29.2)	4 (12.9)	
9 (% of participants)	13 (16.5)	10 (20.8)	3 (9.7)	
10 (% of participants)	22 (27.8)	9 (18.8)	13 (41.9)	
Post‐operative assessment				
Return to sports (RTS)				0.9
No	9 (12.7)	5 (12.5)	4 (12.9)	
Yes	67 (87.3)	41 (87.5)	26 (87.1)	
Time to RTS (months)	7.4 ± 1.7	7.5 ± 1.8	7.2 ± 1.7	0.6
Lysholm	74.8 ± 11.3	74.9 ± 11.5	75.2 ± 11.1	0.4
IKDC	68.9 ± 12.4	67.9 ± 1.8	70.1 ± 9.5	0.2
UCLA	7.6 ± 1.3	7.7 ± 1.2	7.6 ± 1.4	0.7

*Note*: Scores reported as mean ± standard deviation [95% confidence interval).

Abbreviations: BHT, bucket handle tear; IKDC, International Knee Documentation Committee Scores; UCLA, University of California and Los Angeles.

## DISCUSSION

The most important finding from this study was a higher prevalence of ramp lesions on preoperative MRI in patients who underwent a primary ACLR and sustained a post‐operative medial meniscal BHT compared to a lateral meniscal BHT. No ramp lesions were identified or repaired at the index ACLR. This finding suggests that an unrepaired ramp lesion may be associated with a higher risk of post‐operative medial meniscal BHT after a primary ACLR.

A ramp lesion is located at the medial meniscocapsular junction in the posteromedial corner of the knee [12]. A ramp lesion can be easily missed through a standard anterolateral viewing portal because its location is relatively difficult to access with this standard arthroscopy technique [15]. Several techniques have been adapted to improve the visualization and repair of ramp lesions with good clinical results [15, 30, 31]. For example, both a trans‐notch view and switching to a 70° arthroscope may aid in visualizing the posteromedial corner [36]. Further, a posteromedial portal and light debridement of any overlying membrane may facilitate the visualization of a hidden lesion [42]. In our study, a trans‐notch view was only completed in 41 out of 76 cases (54%) of index ACLRs, and a 70° arthroscope was not used during index ACLRs. A needle was placed in the posteromedial space to put the posteromedial capsular tissues on tension in 31 out of 76 cases (41%), but a formal posteromedial portal was not made in any case. It is likely that if these arthroscopic techniques were used on a more regular basis, ramp lesions may have been diagnosed intra‐operatively.

Ramp lesions result in increased tibiofemoral anteroposterior translation and rotational laxity [14, 33]. In a biomechanical study, DePhillipo et al. demonstrated that in ACL‐deficient knees with an associated ramp lesion, the increased rotational laxity was not restored with an isolated ACLR but was restored when concomitant meniscotibial and meniscocapsular repairs were added [14]. However, the clinical utility is unclear due to the lack of a significant clinical improvement after repair of a stable ramp lesion compared to no repair [18, 29]. Further, leaving a stable ramp lesion unrepaired does not seem to influence PROs, return to sport or level of competition [2]. Interestingly, in our study, 21 out of 33 patients who sustained a medial BHT had an unstable ramp lesion [43, 44]. In contrast, only 7 out of 13 patients who sustained a lateral BHT originally had an unstable ramp lesion. In a recent systematic review of 1243 ramp lesions, D'ambrosi et al. found a faster return to sport and no difference in other functional outcomes after a ramp repair compared to no repair [11]. The authors recommended the fixation of unstable ramp lesions and tailoring the treatment of stable ramp lesions based on a case‐by‐case basis [11]. Our study findings align with the notion that the stability of a ramp lesion may be an important difference in whether it requires repair.

MRI has relatively poor sensitivity in the diagnosis of ramp lesions [4, 6, 13, 25, 28]. DePhillipo et al. reported a sensitivity of 48% [13], Arner et al. reported a sensitivity of 53%–84% [4], and in a systematic review and meta‐analysis, Koo et al. reported a sensitivity of 71% [25]. The low sensitivity of MRI in the detection of ramp lesions may be due to the knee being positioned near full extension during the scan, which may result in decreased meniscocapsular separation [6]. Other factors such as scanner resolution, magnet strength, interpreter, slice thickness and the presence of other medial meniscal injuries may also affect MRI detection of ramp lesions [4, 25]. However, secondary signs of a ramp lesion such as a posteromedial tibial bone contusion have recently been identified and improved MRI sensitivity [5]. In our study, no ramp lesions were identified preoperatively on MRI or during diagnostic arthroscopy at the time of the primary ACLR despite approximately half (*n* = 41) having a documented trans‐notch arthroscopic exploration. A combination of the above factors and a generally lower level of awareness and comprehension surrounding ramp lesions during the study collection dates may explain why the ramp lesions were not initially diagnosed. Yet, it is also possible that ramp lesions were overdiagnosed during the retrospective review of the MRIs included in this study. There are previous reports of false positive diagnoses of meniscal tears, specifically posterior longitudinal tears [17, 40]. However, the MRI reviewers in this study used previously validated signs of ramp lesions on MRI in addition to extensive training and experience on the subject matter [13, 27, 43]. Regardless of the preoperative MRI findings, diagnostic arthroscopy during ACLR should include a routine trans‐notch assessment of the posteromedial compartment, with probing of the overlying soft tissue and combined internal rotation of the tibia to minimize the risk of missing an associated ramp lesion.

The effect of a loose but intact ACL graft on knee rotational laxity and subsequent meniscal BHT is an area of growing interest. An ACL‐deficient knee with rotational laxity is at increased risk for subsequent meniscal tear, cartilage damage and eventual osteoarthritis, which can be at least partially mitigated by an ACLR [10, 34, 35, 38, 41]. However, it is unclear if, and to what extent, the risk of subsequent cartilage injury is increased if a loose ACL graft is present that provides some degree of partial rotational stability. In this study, there was no statistical difference between the medial and lateral BHT groups with respect to initial ACL graft integrity or revision ACLR frequency. These results do not indicate that there was an equivalent risk of secondary BHT between intact, loose but functional, and loose and abnormal ACL grafts. Rather, ACL graft integrity and revision incidence did not have a significant association with subsequent BHT. These findings were not the primary outcomes of the study and should be interpreted cautiously. Further investigation into the effect of loose ACL grafts on subsequent meniscal injury is warranted.

## LIMITATIONS

The retrospective nature of this study introduced the potential for recall bias, selection bias, confounding and overdiagnosis of ramp lesions during MRI review. Second, the heterogeneity of surgeons who completed the index ACLRs in this study potentially decreased the internal validity of the results. Further, the small sample size and lack of a control group without associated pathology may have affected the external validity too. However, pooling the results of more than one surgeon reflected a ‘real‐world’ scenario, and this pragmatic approach likely improved the study's generalizability. Fourth, the follow‐up period was only a minimum of two years, and no details about the mechanism of secondary BHT were recorded; therefore, no long‐term conclusions can be made about the consequences of an unrepaired ramp lesion during ACLR with regard to knee longevity or PROs. Fifth, since no time to return to sport was recorded for participants, no conclusions could be made about the effect of an unrepaired ramp lesion on time to return to sport. Finally, although a standard rehabilitation protocol was broadly applied to all patients, it is unlikely that all patients experienced the same rehabilitation protocol while working with different therapists. Different rehabilitation protocols and timing may have impacted the development of secondary meniscal lesions, which could be a further confounder.

## CONCLUSION

After a primary ACLR, an untreated ramp lesion was associated with a post‐operative medial BHT more often than a lateral BHT. Unrepaired ramp lesions may be a risk factor for subsequent medial meniscus BHT after primary ACLR.

## AUTHOR CONTRIBUTIONS

Alexander J. Hoffer analysed and interpreted the patient data and was a major contributor to writing the manuscript. Ahmed Mabrouk, Matthieu Ollivier and Christophe Jacquet contributed to the study design and methodology. All authors read and approved the final manuscript.

## CONFLICT OF INTEREST STATEMENT

Alexander J. Hoffer has received hospitality payments from Arthrex Inc., Smith+Nephew Inc. and ImpactOrtho Inc. The remaining authors declare no conflicts of interest.

## ETHICS STATEMENT

Institutional ethics review board approval and a waiver to consent for retrospective chart review were obtained (PADS21‐177_dgr).

## Data Availability

The data sets used and/or analysed during the current study are available from the corresponding author upon reasonable request.
